# A lower LDL-c/ApoB ratio is associated with an increased prevalence of gallstones in the regional Chinese adult population, according to a retrospective, propensity-matched analysis

**DOI:** 10.3389/fphys.2026.1764905

**Published:** 2026-03-17

**Authors:** Bin Ke, Yongkang Liang, Ying Sun, Xin Dai, Yang Gui, Xueyi Feng

**Affiliations:** 1 Department of Gastrointestinal Surgery, The Second People’s Hospital of Wuhu City (Affiliated Wuhu Hospital of East China Normal University), Wuhu, China; 2 Department of General Surgery, Lu’an Hospital Affiliated of Anhui Medical University (Lu’an City People`s Hospital), Lu’an, Anhui, China; 3 Department of Nursing, The Second People’s Hospital of Wuhu City (Affiliated Wuhu Hospital of East China Normal University), Wuhu, China

**Keywords:** dyslipidemia, LAR, lipid metabolism, metabolic syndrome, prevalence of gallstones

## Abstract

**Objective:**

This study aimed to evaluate the association between the LDL-c/ApoB ratio (LAR) and the prevalence of gallstones in regional Chinese adults.

**Methods:**

We conducted a cross-sectional study involving patients with gallstones who underwent surgical treatment at our hospital from March 2021 to September 2023, as well as e-cases from our medical check-up center during the same period. Participants were divided into gallstone and non-gallstone groups. Data on routine blood and biochemical tests, hypertension, and diabetes mellitus history were collected. The differences between the two groups were analyzed using the chi-square test or Kruskal-Wallis rank sum test. Logistic regression analysis, subgroup analysis, and propensity-matched analysis were performed to assess the relationship between LAR and the prevalence of gallstones.

**Results:**

The study included 801 participants aged over 18 years, of whom 259 had gallstones. After adjusting for relevant confounders, LAR was found to be negatively associated with gallstone prevalence (OR = 0.67, 95% CI: 0.48, 0.95). Propensity-matched analyses confirmed that an elevated LAR remained negatively associated with gallstone prevalence (OR = 0.65, 95% CI: 0.43, 0.98). The dose-response curve indicated a linear negative correlation between LAR and gallstone prevalence.

**Conclusion:**

LAR is negatively associated with the prevalence of gallstones. Although a causal relationship cannot be established, these findings may provide preliminary insights for gallstone prediction in regional Chinese adult populations.

## 1 Introduction

Gallstones are a prevalent benign condition of the biliary system and represent one of the most common diagnoses among emergency room patients presenting with abdominal discomfort, epigastric pain, nausea, vomiting, and loss of appetite (Chen et al., 2012). The primary complications of gallstones include cholecystitis, pancreatitis, and cholangitis (Wang et al., 2020). The prevalence of gallstones varies by region, with estimates suggesting that 10%–20% of the population in Europe and the United States will experience gallstones at some point in their lives (Shaffer, 2005). In mainland China, the prevalence exceeds 8% (Wang et al., 2020), while in Taiwan, it can reach as high as 10% (Chen et al., 2006). A significant number of gallstone cases necessitate surgical intervention, with over 700,000 cholecystectomies performed annually in the United States alone, incurring direct costs exceeding $6 billion (Shaffer, 2005). Complications from gallstones can further escalate healthcare expenses and pose serious health risks, including life-threatening conditions (Wang et al., 2022). Consequently, identifying risk factors for gallstones is crucial for preventing their development.

Gallstones are increasingly recognized as a systemic disease (Di Ciaula et al., 2019) with associations to numerous chronic conditions, including cardiovascular disease (CVD) (Fairfield et al., 2019). Recent large-scale multicenter studies and meta-analyses further support the pivotal role of lipid metabolism abnormalities in gallstone pathogenesis (Zhang et al., 2022). Although the precise mechanism linking gallstone disease with CVD remains unclear, existing research suggests that dyslipidemia might be a contributing risk factor (Fairfield et al., 2019; Rocha and Libby, 2009).

In recent years, the LDL-c/ApoB ratio (LAR) has emerged as an alternative to LDL-c for predicting cardiovascular disease (Drexel et al., 2021), diabetes mellitus (Viktorinova et al., 2021), and kidney disease (Bae et al., 2016). Additionally, LAR has been associated with cardiovascular and all-cause mortality (Xiao et al., 2023). However, its potential use in predicting gallstone prevalence, which shares several common pathogenic factors, has not been explored. Considering that dyslipidemia contributes to both cardiovascular diseases and cholesterol gallstone formation, and that the LDL-c/ApoB ratio reflects LDL particle size and density, we hypothesize that LAR may have predictive value in identifying individuals at risk for gallstones. This hypothesis arises from the shared metabolic disturbances underlying these conditions, particularly lipid metabolism dysfunction. Therefore, this study aimed to assess the predictive value of LAR in relation to the prevalence of gallstones in a Chinese adult population.

## 2 Materials and methods

### 2.1 Study population

864 participants for this multicenter cross-sectional study were recruited between March 2021 and September 2023 from Wuhu Second People’s Hospital and Lu’an Hospital Affiliated of Anhui Medical University. Although the study spanned over 2 years, strict inclusion and exclusion criteria were applied, and all participants underwent standardized data collection and ultrasound examination, minimizing temporal bias. Eligibility criteria included: (i) undergoing ultrasonography; (ii) having complete demographic, anthropometric, and biochemical data, including age, gender, height, weight, blood glucose levels, diastolic blood pressure (DBP), systolic blood pressure (SBP), and liver and renal function indicators. The study was approved by the Ethics Committee of the Second People’s Hospital of Wuhu City (No. 2024-KY-76) and conducted in accordance with the ethical principles outlined in the 1964 Declaration of Helsinki and its subsequent amendments.

Exclusion criteria were: 1. missing gallstone information (n = 28); 2. missing LDL-c/ApoB ratio (n = 35). Ultimately, 801 participants were included in the study, of whom 259 were diagnosed with gallstones.

### 2.2 Data collection and definition

Fasting blood samples were collected from the subjects and analyzed in the laboratory within 1 hour. Blood parameters, including platelets (PLT), neutrophils (NE), lymphocytes (LYM), albumin, globulin, direct bilirubin, indirect bilirubin, ALT, AST, urea, total cholesterol (TC), triglycerides (TG), high-density lipoprotein cholesterol (HDL-C), low-density lipoprotein cholesterol (LDL-C), Apolipoprotein A (Apo A), Apolipoprotein B (Apo B), and cystatin C, were measured using automated flow cytometry and the Roche Cobas 6000 chemistry analyzer for blood glucose testing.

Estimated glomerular filtration rate (eGFR) was calculated from venous blood creatinine levels, incorporating age, gender, weight, and cystatin C levels, using the CKD-EPI or modified CKD-EPI formula (Xia et al., 2023). Relevant data, including age, sex, history of hypertension, and hyperglycemia, were obtained from the patients’ previous examination reports. The LDL-c/ApoB ratio (LAR) was designated as the primary exposure variable.

### 2.3 Definition of gallstones

Ultrasound examinations were performed by an experienced radiologist and served as the primary and routine diagnostic modality for gallstone detection in all eligible participants. CT scanning was not used as a first-line screening tool. It was performed only in cases where ultrasound findings were inconclusive or when abdominal CT imaging had already been conducted for other clinical indications and gallstone information was available from those scans.

### 2.4 Covariates of interest

Potential covariates that may confound the association between LAR index and gallstone prevalence were summarized in multivariate adjusted models. Covariates in our study included sex (male/female), age (years),body mass index (BMI), hypertension, diabetes mellitus, albumin, globulin, direct bilirubin, indirect bilirubin, ALT, AST, urea, eGFR, TC, TG, and HDL-C, taking into account the potential impact of inflammation on gallstones (Fairfield et al., 2019), we calculated the SII index (SII = peripheral neutrophils*platelets/lymphocytes*10^9/L^) and adjusted it.

### 2.5 Statistical methods

Continuous variables are presented as means with standard deviations, while categorical variables are expressed as percentages. The Kolmogorov-Smirnov (KS) test was used to assess whether continuous variables follow a normal distribution. For variables meeting normality assumptions, Student's t-tests were employed for continuous variables, and chi-square tests were used for categorical variables and non-normally distributed data to evaluate differences between groups.

Covariates with a variance inflation factor (VIF) greater than 5, indicating multicollinearity, were excluded from further analysis. Multivariate logistic regression models were applied to investigate the independent associations between the LAR, different LAR tertile groups, and gallstone prevalence, following the guidelines (von Elm et al., 2007). Three models were employed: Model 1 with no covariate adjustment, Model 2 adjusted for sex, age, body mass index (BMI), hypertension, and diabetes, and Model 3 adjusted for all variables.

Smoothed curve fitting using the penalized spline method and generalized additive model (GAM) regression were conducted to further examine the relationship between LAR and gallstone prevalence. Inflection points indicating nonlinear relationships were determined via likelihood ratio tests. Sensitivity analyses were performed using propensity score matching (nearest neighbor matching method, with a caliper set at 0.01 for 1:1 matching, and matching variables including gender, age, and BMI). Statistical significance was set at p < 0.05. All analyses were conducted using Empower software (www.empowerstats.com, X&Y Solutions, Inc., Boston, MA, USA) and R version 4.2.2 (http://www.R-project.org, The R Foundation).

## 3 Results

### 3.1 Baseline characteristics

The baseline demographic characteristics of the participants included in the study are summarized in Table 1. The gallstone group had significantly lower LAR and eGFR levels, suggesting metabolic differences between groups.

**TABLE 1:** Baselines characteristics of participants.

Characteristic	Nonstone formers	Stone formers	P-value
N	542	259	
Age (years)	50.74 ± 12.50	51.17 ± 11.72	0.81
BMI (kg/m2)	24.42 ± 3.77	24.46 ± 3.40	0.51
Albumin (g/L)	44.11 ± 4.10	43.53 ± 4.42	0.12
Globulin (g/L)	27.17 ± 13.52	27.13 ± 5.14	0.38
DBIL (μmol/L)	3.99 ± 1.84	3.83 ± 1.56	0.32
IBIL (μmol/L)	8.26 ± 3.44	7.74 ± 3.04	0.08
ALT (U/L)	25.37 ± 22.29	26.52 ± 23.49	0.50
AST (U/L)	21.92 ± 12.66	21.81 ± 12.74	0.48
Urea (mmol/L)	5.87 ± 3.28	6.00 ± 2.52	0.31
EGFR	93.76 ± 24.97	86.63 ± 26.41	<0.001
TC (mmol/L)	4.57 ± 0.96	4.60 ± 1.00	0.76
HDL.C (mmol/L)	1.24 ± 0.35	1.20 ± 0.31	0.07
TG (mmol/L)	1.70 ± 1.07	1.75 ± 0.94	0.17
GLU (mmol/L)	5.47 ± 1.44	5.28 ± 0.98	0.26
SII	536.34 ± 495.55	548.65 ± 781.50	0.63
LAR	3.33 ± 0.68	2.85 ± 0.69	<0.001
Gender (%)			0.59
Male	316 (57.98%)	156 (60.00%)	
Female	229 (42.02%)	104 (40.00%)	
Hypertension (%)			0.78
Yes	372 (68.26%)	180 (69.23%)	
No	173 (31.74%)	80 (30.77%)	
Diabetes (%)			0.30
No	445 (81.65%)	220 (84.62%)	
Yes	100 (18.35%)	40 (15.38%)	

P-value: obtained by the Kruskal-Wallis rank-sum test for continuous variables and Fisher’s exact probability test for counting.

A lower LDL-c/ApoB ratio (LAR) was associated with a higher prevalence of gallstones.

**FIGURE 1:**
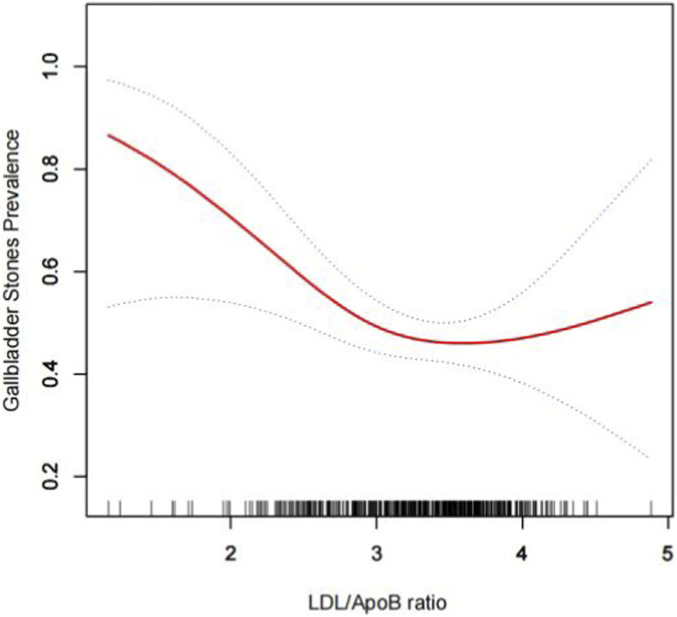
Density dose-response relationship between LAR index with gallstones prevalence. The area between the upper and lower dashed lines is represented as 95% CI. Each point shows the magnitude of the LAR index and is connected to form a continuous line. Adjusted for all covariates except effect modifier.

The variance inflation factor (VIF) for all included variables was below 5, indicating no issues with multicollinearity among the variables. In the fully adjusted model (Model 3), a negative association was identified between the LAR and the prevalence of gallstones (OR = 0.67, 95% CI: 0.48–0.95). This suggests that each unit decrease in LAR is associated with a 33% increased risk of developing gallstones. When LAR was dichotomized based on the median, the high LAR group exhibited a 17% reduction in the likelihood of gallstone prevalence compared to the lowest LAR group (OR = 0.83, 95% CI: 0.58–0.99), as detailed in Table 2. Further analysis using a generalized additive model and smoothed curve fitting confirmed a linear negative correlation between LAR and gallstone prevalence, as illustrated in Figure 1.

**TABLE 2:** Logistic regression analysis between LDL/ApoB ratio with gallbladder stones prevalence.

Characteristic	Model 1 OR (95% CI)	Model 2 OR (95% CI)	Model 3 OR (95% CI)
LAR	0.80 (0.62, 1.03)	0.79 (0.61, 1.03)	0.67 (0.48, 0.95)
Categories			
Lower (1.07–3.36)	1	1	1
Higher (3.36–11.44)	0.89 (0.66, 1.20)	0.89 (0.66, 1.20)	0.83 (0.58, 0.99)

Model 1 was adjusted for no covariates.

Model 2 was adjusted for gender,age and BMI.

Model3 was adjusted for covariates in Table 1.

### 3.2 Sensitivity analysis

**FIGURE 2:**
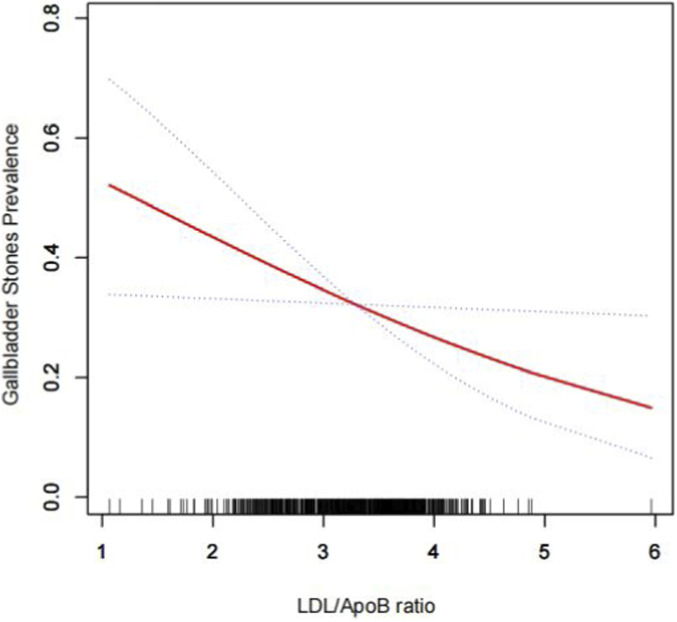
Density dose-response relationship between LAR index with gallstones prevalence after PSM. The area between the upper and lower dashed lines is represented as 95% CI. Each point shows the magnitude of the LAR index and is connected to form a continuous line. Adjusted for all covariates except effect modifier.

Following propensity score matching (PSM), the baseline characteristics of the two groups are summarized in Table 3. Multiple logistic regression analysis demonstrated that the LAR remained significantly negatively associated with gallstone prevalence (OR = 0.65, 95% CI: 0.43–0.98), as shown in Table 4. Further examination of the relationship between LAR and gallstone prevalence after PSM, using generalized additive modeling and smoothed curve fitting, revealed a nonlinear negative correlation (Figure 2). Additionally, a threshold effect was identified with an optimal inflection point value of 2.86 (Table 5).

**TABLE 3:** Baselines characteristics of participants after PSM.

Characteristic	Nonstone formers	Stone formers	P-value
N	245	245	
Age (years)	(245) 47.09 ± 12.17	(245) 51.13 ± 11.86	<0.001
BMI (kg/m2)	(245) 23.55 ± 3.47	(245) 24.56 ± 3.39	<0.001
Albumin (g/L)	(245) 43.82 ± 4.22	(245) 43.74 ± 3.92	0.83
Globulin (g/L)	(245) 26.86 ± 4.80	(245) 27.13 ± 5.14	0.54
DBIL (μmol/L)	(245) 3.88 ± 1.89	(245) 3.84 ± 1.55	0.80
IBIL (μmol/L)	(245) 7.98 ± 3.51	(245) 7.80 ± 3.04	0.54
ALT (U/L)	(245) 22.09 ± 16.19	(245) 26.92 ± 23.97	0.01
AST (U/L)	(245) 20.77 ± 11.65	(245) 22.02 ± 12.99	0.26
Urea (mmol/L)	(245) 5.64 ± 2.35	(245) 5.93 ± 2.35	0.18
UA	(245) 319.85 ± 90.81	(245) 341.97 ± 98.39	0.01
EGFR	(245) 96.90 ± 25.59	(245) 87.16 ± 25.96	<0.001
TC (mmol/L)	(245) 4.46 ± 0.95	(245) 4.61 ± 0.99	0.09
TG (mmol/L)	(245) 1.59 ± 0.88	(245) 1.75 ± 0.93	0.06
HDL.C (mmol/L)	(245) 1.25 ± 0.36	(245) 1.21 ± 0.29	0.08
GLU (mmol/L)	(245) 5.36 ± 1.57	(244) 5.30 ± 0.99	0.61
LAR	(245) 3.33 ± 0.54	(245) 3.23 ± 0.62	<0.05
Gender			<0.05
Male	112 (45.7)	149 (60.8)	
Female	133 (54.3)	96 (39.2)	
Hypertension			0.19
Yes	182 (74.3)	168 (68.6)	
No	63 (25.7)	77 (31.4)	
Diabetes			0.80
No	210 (85.7)	207 (84.5)	
Yes	35 (14.3)	38 (15.5)	

P-value: obtained by the Kruskal-Wallis rank-sum test for continuous variables and Fisher’s exact probability test for counting variables with a theoretical number <10.

**TABLE 4:** Logistic regression analysis between LDL/ApoB ratio with gallbladder stones prevalence after PSM.

Characteristic	Model 1 OR (95% CI)	Model 2 OR (95% CI)	Model 3 OR (95% CI)
LAR	0.73 (0.54, 1.00)	0.75 (0.54, 1.04)	0.65 (0.43, 0.98)

Model 1 was adjusted for no covariates.

Model 2 was adjusted for gender,age and BMI.

Model3 was adjusted for covariates in Table 3.

**TABLE 5:** Threshold effect analysis for association of LDL/ApoB ratio with gallbladder stones prevalence after PSM.

Outcomes	Gallbladder stones
Model 1, β (95% CI)	
Linear effect model	0.73 (0.54, 1.00)
Model 2, β (95% CI)	
Inflection point (K)	2.86
< K	0.26 (0.09, 0.70)
> K	1.10 (0.69, 1.75)
LLR	0.02

## 4 Discussion

For the first time, we investigated the relationship between the LAR, a marker of dyslipidemia, and the prevalence of gallstones using cross-sectional data. Our findings indicate that a lower LAR is associated with a higher prevalence of gallstones in the observed population. These results remained consistent after propensity score matching, underscoring the potential of LAR as a valuable predictor for the development of gallstones.

Numerous studies have reported an association between dyslipidemia and gallstones. Research by Wang (Wang et al., 2020), Banim (Banim et al., 2011), Zhang (Zhang et al., 2022), and others found that HDL-C was negatively correlated with gallstone prevalence. In contrast, studies have shown that triglycerides are positively correlated with gallstone susceptibility (Zhang et al., 2022; Banim et al., 2011; Kato et al., 1992). The association between LDL and gallstone risk has yielded conflicting results in previous studies. Some research found no association (Banim et al., 2011; Kim et al., 2019), while other researchers suggested a protective role for LDL-C (Zhang et al., 2022; Krawczyk et al., 2011). However, more studies have indicated that LDL-C is a risk factor for gallstones (Wang et al., 2020; Chang et al., 2019). These controversial results highlight the need to carefully consider the appropriateness of LDL-C as a predictor of gallstone prevalence. more recent evidence suggests that lipid particle composition and LDL-related metabolic pathways may be more relevant than LDL-C concentration alone (Xiang et al., 2025; Rønborg et al., 2025). Compared to traditional lipid indicators such as LDL-C or HDL-C alone, the LAR integrates both cholesterol content and lipoprotein particle count, potentially reflecting the presence of small dense LDL particles, which are more atherogenic and metabolically active. Unlike LDL-C, which may be misleading in certain metabolic states, LAR offers a more nuanced understanding of lipid-related risks. In our study, LAR consistently demonstrated an inverse association with gallstone prevalence even after adjusting for major covariates and applying propensity score matching.

Current studies have found that the LDL subfraction profile can shift from a healthy “pattern A” (major LDL peaks >255 Å) to a “pattern B” (representing small, dense LDL (sdLDL) particles) (St-Pierre et al., 2005). A significant correlation has been found between a high proportion of sdLDL particles and an increased risk of CVD (Abate et al., 1993; Superko and Gadesam, 2008). Many studies have reported that sdLDL is the most atherogenic parameter, even exceeding LDL, due to its biochemical properties, which allow smaller particles to penetrate the arterial wall more readily and escape receptor-mediated uptake, thereby increasing the risk of atherosclerosis (Superko and Gadesam, 2008; Ivanova et al., 2017). Nuclear magnetic resonance spectroscopy is an effective technique for measuring lipoprotein profiles, yielding results that are less susceptible to the influence of lipoprotein composition. However, the testing conditions are stringent, and the instruments are expensive, making widespread adoption difficult (Blake et al., 2002; Tsai et al., 2004).

The mechanisms linking dyslipidemia and gallstone formation have been increasingly clarified in recent years, particularly within the framework of metabolic syndrome and systemic lipid metabolic dysfunction (Wang et al., 2025). Gallstone disease is now considered part of a broader metabolic disorder characterized by insulin resistance, altered cholesterol homeostasis, and chronic low-grade inflammation. Generally, the formation of cholesterol gallstones depends on cholesterol crystals in the bile, which are associated with an increase in the biliary cholesterol saturation index and are negatively correlated with bile salt levels (Zhang et al., 2022). Research has shown that HDL-C promotes bile acid synthesis (Janowitz et al., 1992) and increases cholesterol solubility in bile, thereby decreasing the cholesterol saturation index (Thornton et al., 1981). Trimethylamine-N-oxide (TMAO) has been found to enhance cholesterol excretion by up-regulating ABCG5/G8, thereby increasing bile secretion of cholesterol in animal experiments, and thus promoting the development of gallstone disease (Chen et al., 2019). TMAO has been shown to regulate lipid metabolism (Schugar and Brown, 2015; Warrier et al., 2015), with elevated levels positively correlating with cardiovascular disease (CVD), atherosclerosis, and metabolic syndrome (Chen et al., 2019). Li et al. found that curcumin could reduce high-fat diet-induced cholesterol saturation in blood lipids and bile in mice and decrease the expression of NPC1L1 and SREBP2 at both mRNA and protein levels, thus preventing the formation of gallbladder stones (Li et al., 2015).

Our study has several strengths. Firstly, we utilized real-world data and applied multi-model logistic regression analyses, enabling comprehensive adjustment for major confounders. Secondly, propensity score matching was performed as a sensitivity analysis, enhancing the robustness and internal consistency of our findings. Future research should further elucidate the physiological mechanisms underlying the association between the LDL-c/ApoB ratio (LAR) and gallstone formation. Large-scale prospective cohort studies are required to clarify temporal and causal relationships, as the cross-sectional design limits causal inference. In addition, detailed assessment of lipid-lowering medication use is necessary to minimize residual confounding and better define the independent contribution of LAR. Mechanistic investigations incorporating advanced lipoprotein subfraction analyses, such as direct quantification of small dense LDL particles, may help determine whether alterations in LDL particle composition contribute to biliary cholesterol supersaturation and gallstone pathogenesis. Furthermore, integrating LAR with metabolic and inflammatory markers may facilitate the development of physiologically informed risk stratification models. However, several limitations should be acknowledged. Due to the cross-sectional nature of the study, causality cannot be established, and the absence of detailed medication data, particularly regarding lipid-modifying therapies, may have influenced the observed associations. Despite these limitations, this study provides novel evidence linking LAR to gallstone prevalence and offers preliminary support for its potential role as a metabolic indicator in gallstone risk assessment.

## 5 Summary

This study suggests that reduced LAR levels were associated with a higher likelihood of gallstone prevalence, indicating that assessment by LAR index levels may benefit gallbladder health. However, further prospective cohort studies are needed to validate our findings.

## Data Availability

The raw data supporting the conclusions of this article will be made available by the authors, without undue reservation.
